# Opportunities and challenges of using social media big data to assess mental health consequences of the COVID-19 crisis and future major events

**DOI:** 10.1007/s44192-022-00017-y

**Published:** 2022-06-27

**Authors:** Martin Tušl, Anja Thelen, Kailing Marcus, Alexandra Peters, Evgeniya Shalaeva, Benjamin Scheckel, Martin Sykora, Suzanne Elayan, John A. Naslund, Ketan Shankardass, Stephen J. Mooney, Marta Fadda, Oliver Gruebner

**Affiliations:** 1 Public and Organizational Health, Epidemiology, Biostatistics, and Prevention Institute, Center of Salutogenesis, University of Zurich, Zurich, Switzerland; 2 Institute for Health Economics and Clinical Epidemiology, The University Hospital of Cologne, University of Cologne, Cologne, Germany; 3 Faculty of Medicine, University of Geneva, Geneva, Switzerland; 4 Infection Control Programme and WHO Collaborating Center on Patient Safety, University of Geneva, Geneva, Switzerland; 5 Division of Public Health Science, Westminster International University in Tashkent, Tashkent, Uzbekistan; 6 Centre for Information Management, Loughborough University, Loughborough, Leicestershire UK; 7 Department of Global Health and Social Medicine, Harvard Medical School, Boston, MA USA; 8 Department of Health Sciences, Wilfrid Laurier University, Waterloo, ON Canada; 9 Department of Epidemiology, University of Washington, Seattle, WA USA; 10 Faculty of Biomedical Sciences, University of Lugano, Lugano, Switzerland; 11 Department of Geography, University of Zurich, Zurich, Switzerland; 12 Epidemiology, Biostatistics and Prevention Institute, University of Zurich, Zurich, Switzerland

**Keywords:** Social media, Big data, Mental health, COVID-19

## Abstract

The present commentary discusses how social media big data could be used in mental health research to assess the impact of major global crises such as the COVID-19 pandemic. We first provide a brief overview of the COVID-19 situation and the challenges associated with the assessment of its global impact on mental health using conventional methods. We then propose social media big data as a possible unconventional data source, provide illustrative examples of previous studies, and discuss the advantages and challenges associated with their use for mental health research. We conclude that social media big data represent a valuable resource for mental health research, however, several methodological limitations and ethical concerns need to be addressed to ensure safe use.

## Introduction

The COVID-19 pandemic has caused detrimental global consequences for public health, healthcare systems, economies, and society in general. It has disrupted day-to-day activities and exposed deep societal inequities as the impacts of the pandemic have been particularly severe among people from socio-economically disadvantaged backgrounds and other marginalized groups [1, 2]. Measures and restrictions including social distancing, closures of non-essential businesses, schools, and national borders were implemented around the world to counter the spread of the virus. While these measures were aimed at “flattening the curve”, they resulted in increases in the risk of adverse mental health consequences [3]. Phenomena such as social isolation, movement restrictions, and economic hardships have significantly contributed to an increase in emotional distress [3, 4]. The pandemic’s global impact on mental health is less immediate and visible than its physical consequences but may prove equally devastating.

Quantifying mental health consequences of a major global event such as the COVID-19 pandemic is extremely challenging. This is in part due to the cost and time associated with collecting and curating data related to mental health that can adequately represent experience on a broader scale. Systematic reviews and meta-analyses of national and multi-national studies can shed light on the changes in mental health symptoms across different regions over the course of the pandemic [5, 6]. However, these methods have inferential challenges including inability to account for heterogeneity in the designs and methodologies of component studies, sampling methods, studied variables, and time-points of data collection [7]. Additionally, surveys often have limited coverage of under-represented groups of the population, such as those living in socio-economically disadvantaged settings, immigrants and refugees, individuals with mental health conditions, and ethnic minority groups [8].

In this commentary, we discuss the role of social media big data in overcoming the existing problems in mental health research. Social media big data can expand our understanding of mental health trajectories throughout the pandemic and across different population groups as they offer broader reach and ability to capture the nuanced social dynamics and enable access to a large amount of timely data “from the field”. This is of utmost importance to identify and mitigate the immediate and long-term adverse psychological consequences associated with global crises such as the COVID-19 pandemic, and to understand and support the development of psychological resilience across populations.

## Opportunities of using social media big data in mental health research

Social media are a digital-based technology that facilitates the sharing of ideas, thoughts, and information through the building of virtual networks and communities. They typically feature user-generated content and personalized profiles. The largest social media networks include Facebook, Instagram, Twitter, YouTube, and TikTok [9]. As of 2022, there were over 4.6 billion social media users globally [10]. The use of social media substantially increased during the pandemic as people had to find ways to fulfill their social needs when physical distancing was imposed [11]. Millions of people every day thus express their thoughts, moods, and emotions via social media platforms [10]. Some users even communicate openly about their everyday struggles with mental health through posts that include written expressions, images, and/or videos. The growing access to smartphones and resulting broader use of social media across the globe creates new opportunities to access data from population groups that may not typically participate in conventional research [12]. Social media data are available in real-time and can be accessed retrospectively, which mitigates the risk of recall bias [13] and offers opportunities for real-time surveillance that could inform preventive measures [14, 15]. They can be collected longitudinally, which facilitates investigation of causal relationships and making predictions about the development of mental health risks [16]. Furthermore, social media often allow to extrapolate information about the users’ sociodemographic characteristics such as gender, age, nationality, or their social network, as well as the geographic location and temporal information [16, 17].

Due to the technological advancements in data processing, it is now possible to conduct accurate and time-efficient analysis of human emotions in big data. Machine learning and Natural Language Processing (NLP) techniques have been shown effective in decoding human emotions and have a great potential to unobtrusively help identifying mental health problems [18]. Machine learning is based on data-driven computational algorithms that can “learn” to recognize data patterns and make predictions. NLP, on the other hand, deals with computational processing of human natural language in text form, such as those found in social media. Advanced spatial epidemiological approaches combining tools such as EMOTIVE [19] or Stresscapes [20], that is, sentiment analysis of basic emotions or stress as found in social media, with spatiotemporal data analysis, allow for example, to track user-based information on the collective emotional state of a given community following the occurrence of significant events [17, 21]. These tools enable the detection of “hotspots” where high levels of negative emotions or stress may indicate a risk for mental health in reaction to local events [22]. Such methods could help to predict mental health development of users in specific regions based on their posts and reactions in real-time. Researchers could thus better understand the dynamics of crises such as COVID-19 and the consequences for mental health in specific subgroups and regions. Governmental and non-governmental organizations could also better adopt appropriate policies and measures, deliver targeted interventions, and allocate limited health care resources to the most affected regions and populations.

## Social media big data and COVID-19

We list some examples of studies that used social media big data, mainly from Twitter, to assess the impact of the pandemic on mental health. A study [23] from the United States (US) analyzed 60 million tweets from the first wave of the pandemic (March–May 2020) and compared these with 40 million tweets from the same period in 2019. They used machine learning classifiers [24] to identify the social media language indicative of mental health problems (anxiety, depression, stress, and suicidal ideation), and found that overall, mental health symptomatic expressions increased by 14% during the COVID-19 period. The increase declined steadily over the period and eventually plateaued, possibly due to habituation effect or the effect of the imposed policy measures. A similar approach followed a study [25] that used machine learning models to identify expressions of stress, anxiety, loneliness, and the overall positive sentiment in a random sample of 5 million tweets/day from March to May 2020 and compared it to the same period in 2019. They identified a significant decrease in overall sentiment, and a significant increase in the expressions of stress, anxiety, and loneliness in 2020 compared to 2019.

Another US study [26] used machine learning and sentiment analysis techniques to assess the changes in social media use in 20 US cities and the shifts in public mood during the first months of the pandemic (January–April 2020). The number of tweets increased over the course of the pandemic and peaked during lockdowns suggesting that people may tend to use social media to cope with social isolation. Similar as in the previous study, there was an overall negative trajectory in the sentiment scores indicating a lower mood than prior to the pandemic. However, the sentiment analysis also revealed an increase in positivity of tweets, which the authors explain by “positivity bias” suggesting that the tweets may not reflect the underlying emotion of the population but rather the language used by media to discuss the topic. Users thus tend to post content that is more positive or optimistic than their true emotions.

Li and colleagues [27] used Twitter data to model spatiotemporal patterns of depressive symptoms associated with the COVID-19 pandemic in the US. They developed an algorithm that combines machine learning with Patient Health Questionnaire [28] lexicon to detect COVID-19 related depressive symptoms during the first months of the pandemic. They found a strong correlation between depressive symptoms and the number of increased COVID-19 cases.

Finally, a cohort study [29] aimed to develop machine learning models for predicting the change in depression and anxiety based on Google Search and YouTube data. They followed a cohort of undergraduate US students (*N* = 49) from January to May 2020 and studied the changes in anxiety and depression scores in relation to data about the students’ use of Google Search and YouTube. Deteriorating depression and anxiety conditions strongly correlated with behavioral changes in Google Search and YouTube use during the COVID-19 pandemic. The study illustrates a potential benefit of machine learning models based on social media big data for evaluating mental health conditions and their development over time.

## Limitations and ethical concerns

Although social media provide an unprecedented access to a wealth of new data sources, their use for mental health research poses several challenges. First, it is challenging to validate social media measures against gold standard assessment tools. For instance, NLP algorithms scanning tweets may indicate the user is experiencing depression symptoms, but it is nearly impossible to assess whether the algorithm accurately reflects standard depression measures at scale and across cultures. Further, social media data restrict the study population to social media users, which may limit the representativeness of the sample and external validity of the investigations. Validity and reliability of the data can also be complicated by automated social media activity (e.g., “bot” accounts) and fake accounts, which can account for a large amount of social media posts [22]. Some of these limitations could be overcome by combining social media data sources with conventional survey-based methods that are prone to other biases (e.g., sampling error, selection bias). Choi and colleagues [30] have demonstrated a successful example of such social media data integration with traditional data collection for the forecasting of weekly suicide fatalities in the US. Finally, as illustrated in one of the studies [26], social media data are prone to various biases such as positivity bias, social desirability bias, or self-idealization which may create an inaccurate image of a user’s mental health status and lead to false conclusions by researchers. Users may engage in some degree of self-idealization, however, research has shown that most social media users express their actual rather than their idealized identities [31].

Apart from methodological challenges, the use of social media big data for mental health research raises important ethical concerns. Although social media can be beneficial for mental health research purposes, the platforms themselves can negatively impact on users’ mental health. Social media contribute to the spread of fake news and hate speech and frequent use of these platforms can lead to feelings of loneliness, depression, and anxiety [32]. Mental health research based on social media data also raises ethical concerns regarding the type of collected data, method of collection and analysis, publication, and any related privacy implications. Although there have been calls to set ethical standards for the use of social media in research [33], there are still no official guidelines in place [34]. Benton and colleagues [35] provide useful recommendations for social media health research regarding aspects such as informed consent, data protection, user interventions, and user identity protection. However, there is still a lack of comprehensive ethical standards in place for social media data use in mental health research. For instance, there remains debate on whether freely accessible social media data should be considered private or public [36]. Information that many would deem sensitive and/or private is therefore frequently shared in ways that was not intended by the user, with a potential for both government and corporate misuse [37]. Mental health research with social media data may involve user interventions such as identification of individuals potentially in need of psychological help. However, the users may have not intended their social media posts to be used for health research purposes, they never signed an informed consent to be part of the research, and they may perceive it as an inappropriate breach of privacy [35]. Due to the stigma around mental health problems, any identification of personal information can be harmful for the individual [37], especially when it involves social media users such as young children or older adults, for whom protection on social media may be insufficient [38].

## Conclusion

Social media big data are a valuable resource for mental health research and may help us to act on acute issues in a timely manner which is of indisputable value for public mental health. Social media data could bring important insights about the scale, severity, and temporal changes of the global mental health burden stemming from the COVID-19 pandemic and future major events. In the long term, they could also contribute to the improvement of access to mental health services, and lead to a more efficient implementation of targeted prevention programs to decrease the incidence of mental disorders in specific areas and populations. Several methodological limitations and important ethical concerns need to be addressed to ensure safe use of such sensitive data.
